# Naturalistic Driving Study in Brazil: An Analysis of Mobile Phone Use Behavior while Driving

**DOI:** 10.3390/ijerph17176412

**Published:** 2020-09-03

**Authors:** Jorge Tiago Bastos, Pedro Augusto B. dos Santos, Eduardo Cesar Amancio, Tatiana Maria C. Gadda, José Aurélio Ramalho, Mark J. King, Oscar Oviedo-Trespalacios

**Affiliations:** 1 Department of Transportation, Federal University of Parana, 81530-000 Curitiba, Brazil; pedroaugusto@ufpr.br; 2 Academic Department of Civil Construction, Federal University of Technology-Parana, 81280-340 Curitiba, Brazil; eduardoamancio@alunos.utfpr.edu.br (E.C.A.); tatianagadda@utfpr.edu.br (T.M.C.G.); 3 National Observatory for Road Safety, 13333-070 Indaiatuba, Brazil; jramalho@onsv.org.br; 4 Centre for Accident Research and Road Safety, Queensland (CARRS-Q), Queensland University of Technology (QUT), Brisbane 5049, Australia; mark.king@qut.edu.au (M.J.K.); oscar.oviedotrespalacios@qut.edu.au (O.O.-T.); 5 Department of Industrial Engineering, Universidad del Norte, Barranquilla, Colombia

**Keywords:** cell phone, driver behavior, human factors, behavioral coding, developing countries, safety

## Abstract

Mobile phone use (MPU) while driving is an important road safety challenge worldwide. Naturalistic driving studies (NDS) emerged as one of the most sophisticated methodologies to investigate driver behavior; however, NDS have not been implemented in low- or middle-income countries. The aim of this research is to investigate MPU while driving and compare the results to those reported in international studies. An analysis of 61.32 h and 1350 km driven in Curitiba (Brazil) showed that MPU lasted for an average of 28.51 s (*n* = 627) and occurred in 58.71% of trips (*n* = 201) with an average frequency of 8.37 interactions per hour (*n* = 201). The proportion of the trip time using a mobile phone was 7.03% (*n* = 201), and the average instantaneous speed was 12.77 km/h (*n* = 627) while using the phone. Generally, drivers spent less time on more complex interactions and selected a lower speed when using the phone. MPU was observed more during short duration than longer trips. Drivers in this study engaged in a larger number of MPU compared to drivers from Netherlands and the United States; and the percentage of trip time with MPU was between North American and European values.

## 1. Introduction

Road crashes are the eighth leading cause of death in the world, which corresponds to 1.35 million deaths. Of these deaths, 80% are concentrated in low- and middle-income countries (LMICs) [1]. Despite the large mortality in the Americas, road safety performance has remained stable during the last seven years, which highlights the challenge of reducing road trauma [2]. Among the risk factors for the occurrence of crashes, in-vehicle distractions caused by engaging in secondary tasks are an increasing road safety concern. Specifically, mobile phone use (MPU) while driving has been identified as one of the main types of distracted driving behaviors. Due to the functionality for everyday life that these devices offer, it is possible that this scenario will worsen as mobile phone devices become more popular and/or evolve technologically [3,4,5]. It is estimated that 87% of the Brazilian population has access to some type of mobile phone—a proportion comparable to that of countries like the United States (89%), Germany (92%) and the United Kingdom (93%)—and 54% of Brazilian users check their device in the first five minutes after waking up, which indicates addiction-like symptoms [6].

The use of mobile phones while driving is also present in LMICs such as Brazil, where the vehicle fleet has grown by more than 60% in the last 10 years [7] and the number of injuries remains high. According to the most recent data (for the year 2017), there were 35,375 traffic-related fatalities in Brazil, equivalent to a rate of 15 fatalities per 100,000 inhabitants [8]. Even though Brazil has laws that prohibit MPU while driving [1], national data on traffic violations within the federal highway system indicates an increase of approximately 40% in the number of fines for MPU while driving between 2015 and 2018 [9]. A survey conducted in 27 Brazilian cities showed that 19.4% of respondents engage in MPU while driving [10].

Understanding driver behavior associated with mobile phone distracted driving is challenging. Official records such as crash data can only provide limited insights regarding the occurrence of distraction, as they can rarely guarantee that a driver was distracted at a particular point [11]. In addition, methodologies such as questionnaires and on-road observations can only provide a limited picture because drivers might exhibit social desirability bias which can result in inaccurate responses to questionnaires or engage in efforts to conceal mobile phone use, which reduces the accuracy of observations. The lack of reliable data on MPU while driving imposes important barriers to evidence-based decision making in LMICs. To address this shortcoming, the prevalence and risks of MPU while driving have been increasingly studied using naturalistic methods in countries such as the United States [12,13,14,15], the Netherlands [16,17], Germany [18], Sweden [19,20], Finland [21], Australia [22] and China [23].

Naturalistic data, however, might present a couple of limitations. In an NDS, it is possible for a single driver to generate multiple MPU episodes, while other drivers generate no episodes, introducing some bias to further analysis [24]. Moreover, results about MPU conversations are not consistent across naturalistic, experimental and epidemiological studies; specifically, texting results are similar between naturalistic and experimental studies but different to epidemiological studies [24]. In addition, it is difficult to predict crash risks and real-world crash profiles considering only the safety critical events detected in NDS [24], even with confidence limits from crash databases being coherent with NDS crash rates [25].

The understanding of driver behavior in Brazil and other LMICs has been limited to survey-based studies [10,26,27], which are subject to desirability and memory biases [15], and driving simulator studies which might have limited external validity [28,29,30]. The aims of this research are to obtain and explore fundamental indicators about MPU while driving through a naturalistic driving study (NDS) and to compare the results to those reported in international studies. The need for this research is justified by the lack of knowledge—in terms of incidence, frequency and characterization—about behaviors that are capable of causing distraction while driving a vehicle, especially distraction from MPU. It has been designed to enable the future replication of procedures on a regional scale or even in other parts of Brazil. In addition, the focus of this research is to survey the fundamental indicators, seeking first to measure more general characteristics on the use of mobile phones while driving, with the expectation that this will raise the interest of researchers and institutions for future research.

## 2. Materials and Methods

### 2.1. The First Naturalistic Driving Study (NDS) in Brazil

**Figure 1:**
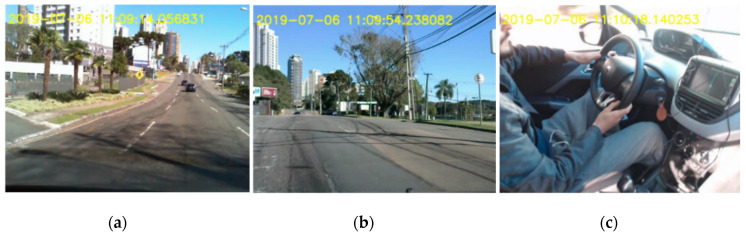
Images collected by cameras: (**a**) frontal left, (**b**) frontal right and (**c**) internal.

The NDS described in this investigation was designed following the principle of “minimum value prototype” with the non-intrusive instrumentation of vehicles, i.e., laptop (Dell Inc., Round Rock, TX, USA), voltage inverter (TechOne, Rio de Janeiro, Brazil), GPS device (Global Positioning System) (Inteform, Sao Paulo, Brazil) and three cameras (LogiTech, Lausanne, Switzerland), one internal and two facing the outside in order to collect images (Figure 1). The laptop, which was positioned in front of the passenger seat, was programmed to activate the cameras and GPS automatically and to collect images. Position and speed data were collected every 1 s. No sound was recorded in order to provide some level of privacy and to avoid inhibiting any conversation related behavior (e.g., having a mobile phone call).

A total of six drivers with a conventional-use vehicle (e.g., home-work trips, leisure trips and routine tasks) were recruited using social networks. The equipment was installed in their own private vehicle—all conventional passenger cars. The participants received an amount equivalent to 100 USD for participating in the NDS. Each driver received the appropriate training in the operation of the monitoring system that had been installed and signed a consent document for privacy and image use. Their ages ranged between 19 and 38 years old, mostly male (4), with driving experience from less than 1 year to 21 years. The oldest vehicle was manufactured in 2002 and the newest in 2013, all with manual gear-transmission and with power ranging from 76 to 165 HP (Table 1).

Each driver was monitored for two weeks, and the data collection period extended from August to November 2019. Participants made a total of 207 trips, with a total duration of 61.32 driving hours and a total distance of 1350 km. The study was undertaken in Curitiba and its metropolitan region, a predominantly urban jurisdiction located in southern Brazil. Data for each driver’s first trip was discarded in order to allow the driver to familiarize themselves with the monitoring system. Only trips that recorded both video and GPS data were considered valid. The periods when the driver was not actually driving were not included in any analysis, i.e., at the beginning of the trip, when the driver turned on the monitoring system without movement of the car; during the trip, when eventually the driver pulled over without turning off the monitoring system; and at the end of the trip, when the driver activated the hand brake but kept the monitoring system on before leaving the vehicle. There were six completely invalid trips, resulting in the total number of valid trips being reduced to 201, the valid trip time to 58.38 h and the total distance to 1303 km.

### 2.2. MPU Analysis

The analysis of the videos taken from the internal camera allowed the identification of visual-manual MPU while driving. The following six MPU behaviors were examined in this study:

- Texting: starting when the driver moves their hand towards the device, then touches the screen with one or both hands several times in a row, and ending when the driver puts the device down and resumes eye contact with the route or engages in another secondary task;
- Calling/voice message: starting when the driver moves their hand towards the device, then uses it for calls or to send/listen to audio in apps, and ending when the driver puts the device down or engages in another secondary task;
- Holding: starting when the driver moves their hand towards the device, then keeps holding the mobile phone, while looking in a different direction than where the device is, and ending when the driver puts the device down or engages in another secondary task;
- In-holder use: starting when the driver moves their hand towards the device, then uses the mobile phone while it is in a holder fixed to the vehicle’s panel/internal windshield screen, and ending when the driver ceases manual contact with the device and resumes eye contact with the route or engages in another secondary task;
- Checking/browsing: starting when the driver moves their hand towards the device, then touches the screen, maintaining visual and/or manual contact with the mobile phone, in order to view information, and ending when the driver puts the device down and resumes eye contact with the route, or engages in another secondary task;
- Other: starting when the driver moves their hand towards the device, then uses it for various purposes other than those described above, such as taking a photo or using the flashlight, and ending when the driver puts the device down and resumes eye contact with the route or engages in another secondary task.

Through manual coding of the videos by a team of trained researchers, a total of 627 MPU events were identified. Five behavioral indicators were extracted: average MPU time (in seconds), I_1_; percentage of trips with MPU (%), I_2_; percentage of time using the mobile phone (%), I_3_; frequency of MPU (use/hour), I_4_; and average instantaneous speed during MPU (km/h), I_5_. For I_4_, a mobile phone use was considered complete each time the driver ceased visual and manual contact with the device. Lastly, I_5_ was the average value of the instantaneous speeds (for every second) during the MPU time that occurred within a valid trip period.

To investigate speed adaptation due to MPU, the average speed was considered between 8 and 10 s before starting the MPU (speed S_1_), the average speed from 8 to 10 s after starting the use (speed S_2_), the average speed from 8 to 10 s before ending the use (speed S_3_) and the average speed from 8 to 10 s after ending the use (speed S_4_). Therefore, the analysis of speed adaptation involved comparing speeds S_1_ and S_2_ and speeds S_3_ and S_4_. Of the 627 MPU events while driving, those that lasted less than 10 s and occurring at a speed of less than 10 km/h (to avoid including MPU while stationary at traffic lights or due to congestion) were not considered in this analysis. Moreover, cases in which S_1_ and S_4_ had values below 10 km/h were not considered. For cases where MPU duration was 10 to 20 s long, the speeds S_2_ and S_3_ had been coincident and centralized over the total period of MPU. Events where the interval between use associated with the S_4_ speed overlapped with the interval associated with the S_1_ speed of the subsequent use were excluded from this analysis.

After applying these criteria, only 17 MPU events remained. The videos of these events were checked, and it was found that all occurred in an urban environment and were influenced by the car-following behavior (that is, by the speed of the vehicle ahead), turning maneuvers or by passing over speed bumps. Thus, there were no MPU events remaining in which speed adaptation could be assessed without the influence of extraneous variables. For this reason, this paper will not evaluate speed adaptation.

Data analysis was conducted using non-parametric methods for comparing the obtained indicator values in terms of categorical variables: in Section 3.2 for comparing I_1_ values according to different types of MPU; in Section 3.3 for comparing I_4_ values for different trip duration categories; in Section 3.4 for comparing I_4_ values according to speed intervals, as well as I_5_ values according to different types of MPU. The limited sample size did not allow for parametric methods.

### 2.3. Benchmarking with International NDS

Although previously published NDS focusing on the use of mobile phones while driving used different methods, the results of the NDS in Brazil were compared with international NDSs selected mainly for their use of indicators comparable to I_1_ to I_5_ (Table 2).

In the United States, data collected from 108 drivers for 6 weeks as part of the Integrated Vehicle-Based Safety Systems Field Operational Test was analyzed [13]. The Netherlands study [16] included data from 21 drivers for a period of 5 to 6 weeks using 5 instrumented vehicles. In Germany, a study was based on data from 49 drivers for a three-month period in Germany, as part of the EuroFOT project (European Field Operational Test) [18]. In Sweden, data collected also in the EuroFOT project from 100 drivers for one year was analyzed [19,20]. In Australia, data from the Australian Naturalistic Driving Study (ANDS) included 377 drivers monitored over 4 months [22]. In the SHRP 2 project (Second Strategic Highway Research Program Naturalistic Driving Study), data from 3262 drivers was examined in the United States [31]. More recently, in the UDRIVE project (European Naturalistic Driving Study), 28 participants were analyzed during a six-month driving period in the Netherlands [17]. In Finland, data from 30 drivers during a period from 63 to 84 days was used [21].

## 3. Results

### 3.1. Characteristics of the Trips Recorded in the Brazilian NDS

**Figure 2:**
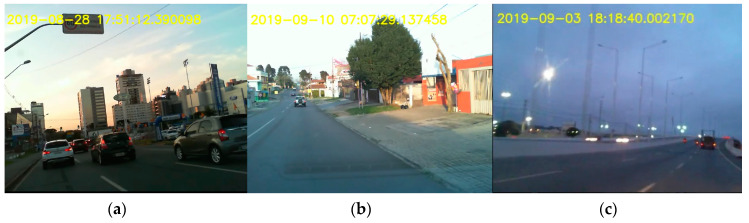
Typical urban roads: (**a**) main, (**b**) local and (**c**) rural urbanized road.

Drivers travelled a total of 1303 km during the monitoring period. 96.95% of this distance was on urban roads and 3.05% on officially rural roads; however, with a high level of urbanization. Figure 2 contains examples of typical urban main road (Figure 2a), local road (Figure 2b) and a rural road with high level of urbanization (Figure 2c). Table 3 shows the individual contribution of each driver to the trips analyzed in this study.

**Figure 3:**
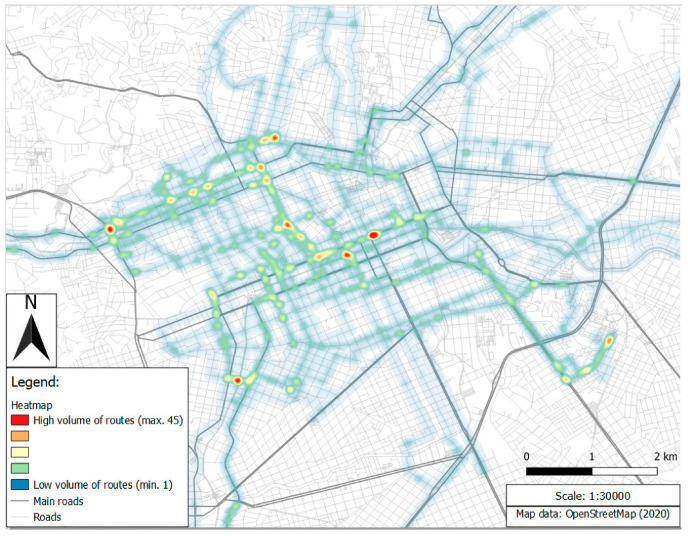
Heat map of the volume of routes and road network in Curitiba main area.

The heat map on Figure 3 shows the concentration of trips in the main area of Curitiba, which is a city with a highly urbanized road network structured following main roads and arterials. The heat map was based on geographical coordinate points collected every second for all drivers. Thus, the visualization represents both traveled distance and travel time densities. Red areas on the map usually consist of intersections between main roads, where drivers tend to spend quite a long time stationary in congestion or at traffic lights. The number of NDS trips on particular road segments ranged from 1 to 45. For scale reasons, Figure 3 visualization covers 98% of the traveled distance considering all drivers.

Table 4 contains information on the share of trips per duration and share of travel time per speed interval. Drivers presented different patterns on trip duration, probably as a function of their commuting distances; for example, while D_2_ had 71.43% of trips lasting more than 30 min, D_4_ and D_6_ did not travel for more than 30 min. According to speed intervals, share of travel time showed more similar distributions between drivers: 63.91% average of the trip time on speed up to 30 km/h, 31.53% average on speed between 30 and 60 km/h and 4.46% average on speed above 60 km/h.

The average number of trips on weekdays per driver was 2.78. Of all the trips, 84.08% were from Monday to Friday, while on weekends the average number of trips per driver was 1.28 (53.96% lower than on weekdays). The start times of trips were concentrated in the afternoon period, from 5 to 6 p.m. (20.71% of the trips), from 7 to 8 p.m. (10.65%) and from 6 to 7 p.m. (8.28%). Morning had lower concentrations, i.e., 8.28% between 7 and 8 a.m., 7.69% between 9 and 10 a.m., and 4.73% between 8 and 9 a.m. The incidence of MPU according to the trip duration showed that 61.98% of the trips with up to 15 min duration included MPU; however, for trips lasting between 15 and 30 min, this proportion decreased to 10.64%, and for trips lasting more than 30 min the proportion was 9.09%.

### 3.2. Average Time That Driver Spent Using Mobile Phone and Type of Use

**Figure 4:**
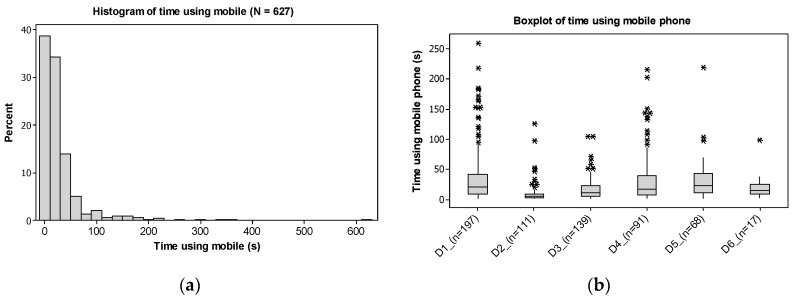
Mobile phone usage time histogram (**a**) and boxplot (**b**) representation.

The average time spent engaged in visual-manual MPU, I_1_, was 28.46 s (*SD* = 47.71 s) based on the 627 events. In the present study, the range of time engaged in MPU varied from 2 s to 10.48 min. While usage duration less than 10 s represents 38.76% of the events (Figure 4a), durations that were between 10 and 50 s represented 48.17% of events. All drivers used their mobile phones, with an average time ranging from 10 s, for D_2_ (driver 2), and 38 s for D_5_ (Figure 4b). In order to present the boxplot of Figure 4b on an appropriate scale, four outliers of MPU duration were removed, namely, 629 s for D_5_, 367 s for D_4_, 344 s for D_3_ and 297 s for D_1_.

The most common type of use was checking/browsing the device at 46.11% of the time spent, followed by simply holding the mobile phone (without eye contact) for 21.92% and 14.46% for making/receiving calls, including listening to and recording audio messages (Table 5). The use of the mobile phone to type messages corresponded to 7.39% of the total time of use. Calling/voice message demanded an average time of 63.05 s (*SD* = 73.50 s), the longest among the types of use; the average texting task duration was 20.03 s (*SD* = 20.15 s) and the in-holder use 11.07 s (*SD* = 14.90). In addition, Wilcoxon signed rank tests indicated that calling/voice message duration is greater than checking/browsing (*W* = 13,525.0, *p* < 0.001), in-holder use (*W* = 6168.5, *p* < 0.001) and texting durations (*W* = 2902.0, *p* < 0.001); checking/browsing duration is greater than in-holder use duration (*W* = 123,885.5, *p* < 0.001); and texting duration is greater than in-holder use duration (*W* = 13,511.0, *p* < 0.001).

### 3.3. Average Percentage of Trips with MPU, Average Percentage of Trip Time with MPU and Frequency of Use

The average percentage of trips with MPU while driving (I_2_) was 58.71% (*SD* = 25.80%), varying between 29.73% (D_6_) and 93.10% (D_1_). Table 6 contains the values of means, standard deviation, minimum, maximum and quartiles for I_3_ (mobile phone usage time percentage) and I_4_ (frequency of use) considering the 201 valid trips and considering the 627 MPU events for I_5_ (instantaneous average speed during MPU).

**Figure 5:**
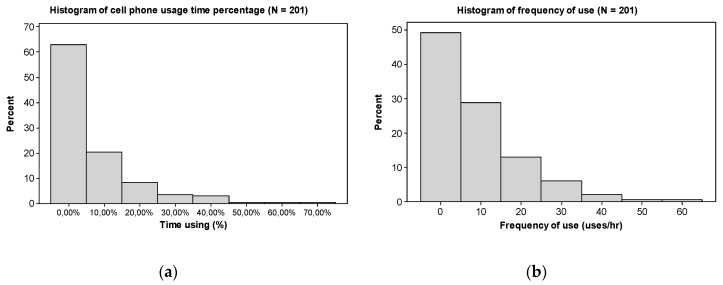
Histogram of percentage of time using cell phone (**a**) and frequency of cell phone use (**b**) (for all participants).

In general, low I_3_ values (<5%) are more common since they correspond to 63.18% of the trips (Figure 5a). For higher values of I_3_, the share of participation reduces, for instance, to 20.40% for I_3_ between 5 and 15% of the time and to 8.46% for I_3_ between 15 and 25%. On average, 8.37 occurrences of MPU per hour (*SD* = 10.69 uses/h) were identified (I_4_), equivalent to one MPU event every 7.17 min. The histogram in Figure 5b shows the distribution of I_4_ values, where an amount of up to 5 uses/h is observed in 49.25% of trips and an I_4_ value between 5 and 1_5_ uses/h in 28.86% of trips. The comparison between frequencies of MPU in different trip durations showed that there is some influence of trip duration on engagement in MPU while driving. Wilcoxon signed rank tests showed that the frequency of MPU in trips between 0 and 15 min was lower than in trips between 15 and 30 min (*W* = 8743.0, *p* < 0.001), 30 and 45 min (*W* = 7909.5, *p* < 0.001), 45 and 60 min (*W* = 7607.0, *p* = 0.002) and longer than 60 min (*W* = 7587.0, *p* = 0.015).

### 3.4. Average Instantaneous Speed While Using the Mobile Phone

**Figure 6:**
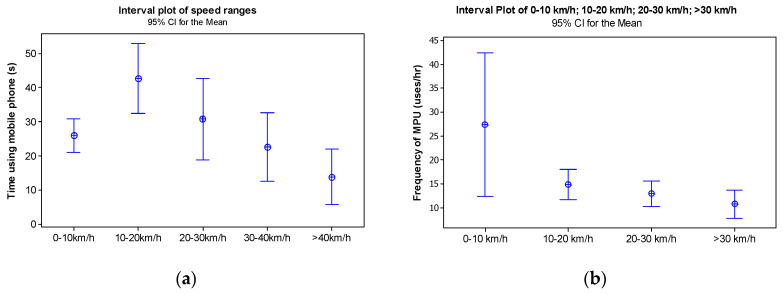
Interval plot graphs for average time (**a**) and frequency of MPU (**b**) by speed category.

The average instantaneous speed during MPU (I_5_) is 12.77 km/h (*SD* = 16.38 km/h). Based on the association between the indicators of frequency of use and percentage of time using the mobile phone at a given speed, confidence interval graphs were constructed showing the means (Figure 6). In Figure 6a it can be seen that the average MPU time was lower for the speed range 0 to 10 km/h (when there is a greater number of cases), while it increases for the speed range 10 to 20 km/h, and it starts to reduce continuously as speed increases.

When it comes to the frequency of MPU, a higher mean (but with a wide standard deviation) was identified for the speed range 0 to 10 km/h (Figure 6b), for which the number of cases using the mobile phone is substantially greater. It can be noted that the average values of I_5_ and the variation around the mean reduce to speed ranges between 10 and 20 km/h and between 20 and 30 km/h, when the number of MPU cases reduces. Wilcoxon signed rank tests show statistically significant differences between frequencies of MPU according to speed intervals: Frequencies of MPU are higher for speeds between 0 and 10 km/h in comparison to speeds between 10 and 20 km/h (*W* = 253.0, *p* = 0.018), between 20 and 30 km/h (*W* = 327.5, *p* = 0.005) and higher than 30 km/h (*W* = 113.0, *p* = 0.004).

The types of use that tend to demand greater visual-manual contact, which causes a higher level of distraction (such as typing messages), were performed at a lower average speed of 10.57 km/h (*SD* = 12.30 km/h). On the other hand, the average speed while engaged in in-holder use (16.45 km/h, *SD* = 15.39 km/h) and calling/voice message (17.28 km/h, *SD* = 14.80 km/h) secondary tasks was higher. Wilcoxon signed rank tests confirms these assumptions, since speed while calling/voice message is greater than checking/browsing (*W* = 11,413.0, *p* = 0.002) and texting speeds (*W* = 2642.0, *p* = 0.003); checking browsing speed is lower than use on-holder speed (*W* = 108,223.0, *p* < 0.001); and texting speed is lower than in-holder use speed (*W* = 16,908.0, *p* = 0.009).

## 4. Discussion

This paper describes the first NDS performed in Brazil, including six drivers below 40 years old resulting in 58.38 driving hours and a total distance of 1303 km available for analysis, mostly traveled in urban area. In this study, MPU while driving lasted 28.46 s on average with 46.11% of that time being used to “check/browse” on the phone. MPU occurred in 58.71% of trips, the average frequency of use was 8.37 uses/h, the percentage of trip time using the mobile phone was 7.03%, and the average instantaneous speed was 12.77 km/h while engaged in MPU. The confirmation of the large prevalence of MPU while driving in Brazil is very concerning because previous research has consistently shown that visual-manual interactions with a mobile phone while driving such as texting or browsing impairs performance of the driving task [30,31,32,33,34]. Consequently, MPU while driving can be associated with an increased risk of crashing [31,35,36]. The findings from this study and the associated risks of MPU while driving highlight the need to urgently develop interventions to reduce MPU while driving in Brazil.

### 4.1. MPU Duration, Trip Length and Driving Speed Analysis

Comparisons between the duration of different MPU behaviors were conducted. The results showed a difference in average duration of MPU and average speed using the phone while driving according to the type of use (texting, calling/voice message, in-holder use and checking/browsing). Drivers tend to spend less time using the mobile phone for complex MPU such as texting, which involved looking at the screen of the device and typing, as opposed to browsing/checking which does not require typing. The large difference in average duration of in-holder MPU (11.07 s) and calling/voice message MPU (63.05 s) is consistent with the assumptions that (i) drivers perceive that talking and voice messaging do not deviate much attention needed for safe driving, so they tend to spend more time on these activities; and (ii) in-holder use tends to be more uncomfortable, thus mobile phone use tends to be brief. These behaviors can be interpreted as a risk-compensatory strategy of drivers to prevent excessive workload from the secondary task. Previous experimental research [37] has demonstrated that drivers actively seek to reduce or manage the additional workload produced by mobile phone interactions. Specifically, drivers make tactical decisions to reduce workload by selecting types of MPU that are shorter and easier to self-pace in case the driver needs to suddenly focus all of their attention on the driving task again.

The results from this study also show that MPU while driving increases during longer trips and lower average driving speeds. The associations between length of the trip and MPU further confirm associations between driver states such as boredom with engagement in mobile phone distracted driving. Previous research has shown that mobile phones are utilized by drivers as a source of stimuli to overcome boredom [38]. Drivers have generally also been observed reducing their speed in order to facilitate MPU. This finding is well established in the scientific literature through self-reports [39,40], experiments [41,42,43] and naturalistic studies [15,44]. It has been demonstrated that this change in the driving speed is used by drivers to reduce crash risk [45].

### 4.2. Comparisons with International Studies

The values found in this study resemble those of other international NDS, highlighting the seriousness of MPU. International comparisons indicate the average time spent engaged in visual-manual MPU (I_1_) of 28.46 s (*SD* = 47.71 s) is similar to the average time of 31 s found in a Netherlands naturalistic study [16] and the average time of 30 s found in a North American naturalistic study carried out in Detroit, MI [13]. For the German study, the I_1_ value was 46 s [18]. This result is not surprising since research has consistently showed that drivers in LMICs show the same or more propensity to engage in risky driving behaviors due to a wide number of systematic factors such as insufficient legislation, lack of effective police enforcement, poor education and training, etc. [46]. The average percentage of trips with MPU while driving (I_2_) of 58.71% (*SD* = 25.80%) was much higher than the value of 13% found in a Swedish NDS [20], higher than that found in two studies in the Netherlands, an older one with 40% [16] and a more recent one with 32% [17]. However, the present Brazilian result (I_2_) is similar to the results of the German NDS that found MPU in 56% of the trips [18].

The Brazilian value of 7.03% of time using the mobile phone (I_3_) is similar in comparison to the Australian value of 7% [22]. Although the average value found in this research for I_3_ is lower than the most recent Netherlands study, 9.2% [17], it is higher than the Swedish value, 0.63% [19], the North American values, 2.3% [13] and 6% [31], as well as the older Netherlands study with 4% [16]. On average, 8.37 MPU occurrences of use per hour (*SD* = 10.69 uses/h) were identified (I_4_), equivalent to one MPU event every 7.17 min. The 8.37 MPU occurrences of use per hour (*SD* = 10.69 uses/h) identified (I_4_) is nearly twice the frequency of 4.2 uses/h calculated for the Netherlands study [16] and more than three times the value of 2.5 uses/h from the North American study [13]. Finally, although driving speed is strongly influenced by the operational characteristics of the environment driven and a direct comparison is very difficult [47,48], the average instantaneous speed during MPU of 12.77 km/h (*SD* = 16.38 km/h) is much lower than the value (34 km/h) obtained in the Finnish study [21].

### 4.3. Limitations of the Study

The first NDS in Brazil had limited scope with a small sample size, since drivers (all under the age of 40) from one city in Brazil may not represent all Brazilian drivers or even less all drivers in LMICs. Another potential limitation is selection bias; as just certain types of drivers would have been attracted to participate in the study due to the financial rewards or to privacy concerns. Future research is needed to strengthen the generalizability of the findings and to overcome limitations. Increasing the geographical coverage and sample size of the current study is one option to increase generalizability of the findings. In addition, a more diverse group of participants would enable identification of human factors and age-specific determinants of driver behavior. Moreover, evaluation of speed variation due to MPU might be possible in a larger scale NDS.

The participants were not told that mobile phone use while driving was going to be specifically registered. However, it was clear to drivers that they were participating in driver behavior research, in which one would probably expect that mobile phone use would be monitored. Drivers were told to behave exactly how they would behave if they were in their natural driving conditions. It was possible to identify that drivers were performing typically private behaviors (for example, scratching or applying make-up), that is, actions usually performed alone or in a very familiar environment.

## 5. Conclusions

The current study offers unique insights into driver behavior that have not been reported in previous research in Brazil. The development of low-cost solutions for conducting behavioral research, like the one presented in this manuscript, contributes to the development of road safety knowledge and traffic behavior in LMICs. Naturalistic driving studies are a viable alternative for the survey of road user behavior in LMICs. Overall, these findings show that the results from this investigation resemble previous research findings, which in turn provides evidence for the validity of this Brazilian NDS. Therefore, the Brazilian NDS is a low-cost and valid methodology to investigate MPU while driving.

The present research identified evidence of the strategies used for drivers in Brazil to compensate for excessive workload due to mobile phone use while driving. Drivers spent less time on the mobile phone for complex MPU, such as texting, as opposed to less complex tasks like browsing/checking. Moreover, it was observed that MPU when driving increases during longer trips and lower average driving speeds. Drivers in this study also showed a larger number of engagements in MPU than values from countries such as the Netherlands and the United States. On the other hand, the percentage of MPU time obtained in this research is between North American and European values. These findings may be the result of cultural, legislative and enforcement differences between high-income versus low- and middle-income countries.

## Figures and Tables

**Table 1:** General info of participants.

Driver	Age	Gender (M/F) ^1^	Driving Time Experience (Years)	Car Model	Car Model Year	Unit of Power (HP) ^2^
D_1_	31	F	10	Chevrolet/Prisma	2012	97
D_2_	38	M	<1	Renault/Scenic	2009	115
D_3_	19	M	<1	VW/Fox	2010	104
D_4_	23	M	4	GM/Zafira	2002	116
D_5_	38	F	21	VW/Fox	2013	76
D_6_	25	M	7	Citröen/DS3	2012	165

^1^ M, Male; F, Female; ^2^ HP, Horsepower.

**Table 2:** International studies used for results comparison.

Country	Authors	I_1_^1^	I_2_^2^	I_3_^3^	I_4_^4^	I_5_^5^
United States	Funkhouser and Sayer (2012) [13]	✓		✓	✓	
Netherlands	Christoph, Nes and Knapper (2013) [16]	✓	✓	✓		
Germany	Metz, Landau and Just (2014) [18]	✓	✓			
Australia	Young et al. (2019) [22]		✓			
Sweden	Tivesten and Dozza (2014) [19]		✓			
	Tivesten and Dozza (2015) [20]			✓		
United States	Dingus et al. (2016) [31]		✓			
Netherlands	Christoph, Wesseling and van Nes (2019) [17]		✓	✓		
Finland	Kujala and Mäkelä (2018) [21]					✓

^1^ I_1_, average time using mobile phone; ^2^ I_2_, percentage of trips with MPU; ^3^ I_3_, percentage of time using the mobile phone; ^4^ I_4_, frequency of MPU; ^5^ I_5_, average instantaneous speed during MPU.

**Table 3:** Total number of trips and number and proportion of trips using a mobile phone by each driver.

Driver	Total Number of Trips	Share of Total Trip (*SD* = 8%)	Traveled Distance (km)	Share of Total Traveled Distance (*SD* = 4%)	Share of Traveled Distance in Urban Roads (%)	Share of Traveled Distance in Highways (%)
D_1_	29	14.29	227.610	17.47	100.00	0.00
D_2_	14	7.88	263.557	17.85	96.10	3.90
D_3_	17	8.37	207.399	15.73	99.86	0.14
D_4_	48	23.65	235.228	17.73	100.00	0.00
D_5_	56	27.59	286.997	21.43	89.13	10.87
D_6_	37	18.23	129.752	9.79	100.00	0.00
Total	-	100%	1303	100%	-	-

**Table 4:** Share of trips per duration and share of travel time per speed.

Driver	Share of Trips per Duration (%)			Share of Travel Time per Speed (%)		
	0–15 min	15–30 min	Above 30 min	0–30 km/h	30–60 km/h	Above 60 km/h
D_1_	13.79	51.72	34.48	71.04	26.74	2.23
D_2_	7.14	21.43	71.43	67.04	30.62	2.34
D_3_	23.53	41.18	35.29	57.18	34.17	8.66
D_4_	75.00	25.00	0.00	60.21	34.49	5.31
D_5_	75.00	12.50	12.50	63.02	32.62	4.36
D_6_	91.89	8.11	0.00	64.99	30.56	4.45
Mean	47.73	26.66	25.62	63.91	31.53	4.46

**Table 5:** Different type of use regarding the mobile phone by total usage time and I_1_.

Type	Usage Time (s)	Usage Time (%)	I_1_ (s) 95% CI [LL–UL] ^3^
Checking/browsing	8244	46.11	20.31 [23.20–17.41]
Holding	3919	21.92	37.68 [28.57–46.79]
Calling/voice message	2585	14.46	63.05 [39.84–86.26]
On-holder	1639	9.17	11.07 [8.65–13.50]
Texting	1322	7.39	35.73 [15.98–24.98]
Other	93 ^1^	0.52	-
NPI (Not Possible to Identify)	76 ^2^	0.43	-

^1^ Only 7 observations; ^2^ Only 3 observations; ^3^ I_1_, Average usage time; CI, Confidence interval; LL, Lower limit; UL, Upper limit.

**Table 6:** Descriptive Statistic for I_3_, I_4_ e I_5_ values.

Statistic Parameter	I_3_^1^ (%) *n* = 201	I_4_^2^ (uses/h) *n* = 201	I_5_^3^ (km/h) *n* = 627
Mean	7.03	8.37	12.77
Standard deviation	11.16	10.59	14.14
Minimum value	0.00	0.00	0.00
1º quartile (Q_1_)	0.00	0.00	0.92
Median	1.90	5.26	8.21
3º quartile (Q_3_)	8.89	13.03	22.08
Maximum value	65.75	57.75	61.96

^1^ I_3_, percentage of time using the mobile phone; ^2^ I_4_, frequency of MPU; ^3^ I_5_, average instantaneous speed during MPU.
